# Risk factors for mortality of children with zoonotic visceral leishmaniasis in Central Tunisia

**DOI:** 10.1371/journal.pone.0189725

**Published:** 2017-12-29

**Authors:** Khaled Ben Helel, Mohamed Ben Rejeb, Zakia Habboul, Nizar Khattat, Houssain Mejaouel, Houyem Said-Latiri, Belhassen Kaabi, Elyes Zhioua

**Affiliations:** 1 Pediatric Department of University Hospital of Kairouan, Kairouan, Tunisia; 2 Department of Prevention and Care Safety, University Hospital of Sahloul, Sousse, Tunisia; 3 Pasteur Institute of Tunis, Tunis, Tunisia; Academic Medical Centre, NETHERLANDS

## Abstract

**Background:**

Zoonotic visceral leishmaniasis (ZVL) caused by *Leishmania infantum* is endemic with an epidemiological profile of a paediatric disease in Tunisia. In the context of a high fatality rate, identifying risk factors for in-hospital mortality in children treated for ZVL is of major epidemiological importance.

**Design:**

A retrospective (case-control) study included 230 immuno-competent children diagnosed and confirmed with primary ZVL in the paediatric department of the University Hospital of Kairouan between 2004 and 2014. Forty-seven per cent (47%) were children under 18 months of age, and with a male ⁄ female ratio of 1.01:1.

**Results:**

The overall case-fatality was 6% (n = 14). The risk factors for in-hospital death identified by a multivariate analysis were: bleeding at admission (OR = 25.5, 95% CI: 2.26–287.4; p = 0.009), white cell count less than 4000/mm3 (OR = 5.66, 95% CI: 1.16–27.6; p = 0.032), cytolysis (OR = 28.13, 95% CI: 4.55–173.6; p < 0.001), and delay between onset of symptoms and admission ≥ 15 days (OR = 11, 95% CI: 1.68–72; p = 0.012).

**Conclusion:**

The results strongly suggest that paediatric patients admitted 15 days after onset of symptoms, with bleeding, white cell counts below 4,000/mm^3^, and cytolysis at admission should be considered severe cases and subsequently, they are at high risk of mortality. A better understanding of factors associated with death of children from ZVL may contribute to decrease mortality.

## Introduction

Zoonotic Visceral Leishmaniasis (ZVL) is a vector-borne zoonotic disease caused by *Leishmania infantum*, which is transmitted by the bite of phlebotomine sand flies. ZVL can affect both humans and canines, and it is considered by the WHO to be one of the most important neglected tropical diseases, affecting about 0.5 million people per year [1]. ZVL is widespread in South and Central America, North Africa, Southern Europe, Middle- and Far-Eastern countries, and is strongly correlated with poverty [2–3]. In the Western Mediterranean basin, sand flies of the subgenus *Larroussius*, mainly *Phlebotomus perniciosus*, is the main vectors of ZVL [4, 5]. In Tunisia, the zymodeme MON-1 is responsible for the majority of human and canine cases [6–10]. The domestic dog is the main reservoir host for *L*. *infantum* [10].

ZVL is a peridomestic disease endemic mostly in rural areas, affecting families with low social and economic status [3]. ZVL is systemic and results in the death of an infected individual if left untreated. No effective vaccine against ZVL is available for humans [11], and treatment is based on chemotherapy. The only treatment of ZVL available in Tunisia is based on meglumine antimoniate (Glucantime®) [12–13], which has been used for more than 40 years. In addition to its toxicity to humans [14–15], resistance of *L*. *infantum* to this drug has been confirmed [16].

Until the 1980s, cases of ZVL in Tunisia were limited to the northern humid, sub-humid and semi-arid areas [17–20]. However, more recently ZVL has become endemic in arid areas located in Central Tunisia following the report of several autochthonous cases [13, 21–23]. In Tunisia, ZVL is responsible for considerable child morbidity and mortality with an estimated incidence between 100 and 160 cases per 100,000 inhabitants [2], affecting mostly children less than 5 years old [8, 12, 21–23]. In Tunisia, the mortality rate is 6% [12], and therefore, ZVL is considered a major public health problem. This study aims to identify risk factors associated with death of Tunisian children diagnosed with ZVL.

## Methods

### Ethical statement

The study was approved by the Ethical Review Board of the Regional Directory of Health of the governorate of Kairouan (see supplementary materials section S1 Fig). Informed consent was obtained from the study participants upon signing an explained agreement by their parents (see supplementary materials: S2 Fig). Data were analyzed anonymously.

### Study design and population

A retrospective study concerning a cohort of children and adolescents with up to 15 years of age admitted for ZVL treatment between 2004 and 2014 was carried out at the paediatric department of the University Hospital of Kairouan located in Central Tunisia, a referral center for the treatment of ZVL. Cases were defined as patients younger than 15 years with confirmed diagnosis of ZVL, who died during hospitalization. The survivors were considered as controls.

### Diagnostic procedures

The ZVL diagnosis was performed according to the recommendations of the laboratory of the Ministry of Health. Parasitological diagnosis was performed based on detection of amastigotes in Giemsa stained bone marrow smears, and by isolation of promastigotes from culture media (McNeal, Novy & Nicolle medium: NNN). Immunological tests were performed by using immunofluorescence antibody test (IFAT) and/or polymerase chain reaction (PCR). The diagnosis is considered confirmed if at least one laboratory test, parasitological or immunological test is positive.

### Data collection

The medical records of all patients were reviewed and abstracted by using a standardized data collection tool for demographic information and data related to risk factors (age, gender, delay between onset of symptoms and admission, pallor, splenomegaly, size of splenomegaly, hepatomegaly, nutritional status, bleeding, co-infections, leucopenia <4000 cells/mm^3^, haemoglobin <6g/dL), platelet count <50000/mm^3^, cytolysis and hemophagocytic syndrome). Hypotrophy or failure to thrive is often defined as weight per age that falls below the 5th percentile on multiple occasions or weight deceleration that crosses two major percentile lines on a growth chart [24]. Laboratory variables were categorized according to the following cut-off points: (i) leucopenia (<4,000/mm^3^); (ii) deep anaemia (haemoglobin <6 g/dL); (iii) deep thrombocytopenia (platelet count <50,000/mm^3^) and cytolysis (ALT > 80 UI/L and/or AST > 70 UI/L).

A diagnosis of hemophagocytic syndrome was made when five or more of the following criteria were fulfilled: 1) fever; 2) splenomegaly; 3) cytopenia affecting at least two of the three lineages in the peripheral blood (haemoglobin < 90 g/L, platelets <100×109/L, neutrophils <1.0×109/L); 4) hypertriglyceridemia and/or hypofibrinogenemia (fasting triglycerides ≥3.0 mmol/L or ≥3 SDs, fibrinogen ≤1.5 g/L or ≤3 SDs); 5) ferritin ≥500 μg/L; 6) soluble CD25 (soluble IL-2 receptor) ≥2400 U/mL; 7) low or absent NK cell activity; and/or 8) hemophagocytosis in the BM, spleen or lymph nodes [25].

### Statistical analysis

Data analysis was performed using GNU PSPP version 0.10.4 and the R software for statistical computing version 3.2.4 for Windows. Continuous variables were described as means ± standard deviations. Categorical variables were summarized with absolute and relative frequencies. To compare percentages, we used Chi square test. Means were compared using Student t-test. Potential risk factors with p-values of 0.20 or less in the initial univariate logistic regression were included in the multivariate binary logistic regression models. The model was reduced by means of manual backward elimination. Statistical significance was set at p-value ≤ 0.05.

Multiple corresponding analysis (MCA) was performed using the R package FactoMiner to detect potential multivariate association between categorical or categorized clinical variables and the mortality by ZVL.

## Results

Overall, 230 children with ZVL were included. The mean age of patients was 25.1 ± 26.2 months. Of these, 50.4% were males and 49.6% females. The mean delay between onset of symptoms and admission was 28 ± 32.4 days. The most common symptoms were fever (94%), splenomegaly (87.8%) and pallor (71.7%). Furthermore, hypotrophy was found in 18.3% of children. Seven patients presented bleeding upon admission. Co-infections (viral and bacterial) were documented in 51 children (22.2%). Biological tests showed profound anemia (Hb< 6g/dL), leucopenia, deep thrombocytopenia (platelet count <50,000 cells/mm3) and cytolysis respectively in 21.7%, 59.1%, 20.4% and 71.2%. Hemophagocytic syndrome was identified in 59 patients. During this study, 14 children with ZVL died. The mortality rate was 6.1%. Table 1 illustrates the characteristics of the study population.

**Table 1:** Characteristics of children with ZVL.

Characteristic		Number (n)	Percentage (%)
Age (months)	< 18	108122	4753
	≥ 18		
Gender	Male Female	116114	50.449.6
Delay between onset of symptoms and admission (days)	< 15 ≥ 15	14090	60.939.1
Pallor	YesNo	16565	71.728.3
Splenomegaly	YesNo	20228	87.822.4
Size of splenomegaly	< 2≥ 2	65165	28.371.7
Hepatomegaly	YesNo	102128	44.355.7
Nutritional status	Eutrophic Hypotrophic	18842	81.718.3
Bleeding	YesNo	7223	397
Co-infections	Yes No	51179	22.277.8
Leucopenia	Yes No	13694	59.140.9
Haemoglobin (g/dL)	< 6≥ 6	50180	21.777.3
Platelet count (/mm^3^)	<50,000≥ 50,000	47183	20.479.6
Cytolysis	YesNoNot done	1862222	71.29.69.6
Hemophagocytic syndrome	YesNo	59171	25.774.3
Death	YesNo	14216	6.193.9

In the univariate analysis, mortality was associated with bleeding (p = 0.005), Hb < 6g/dL (p-value = 0.021), cytolysis (p <10–3), hemophagocytic syndrome (p-value = 0.014) and treatment intolerance (p-value = 0.006) (Table 2).

**Table 2:** Univariate analysis of risk factors of mortality in children with ZVL.

Factor	Died (%) (n = 14)	Survivors (%) (n = 216)	OR [95%CI]	p
**Age**	8 (7.4) 6 (4.9)	100 (92.6) 116 (95.1)	1.55 [0.52–4.6]	0.43
< 18 months≥ 18 months				
**Gender**	8 (6.9) 6 (5.3)	108 (93.1) 108 (94.7)	1.33 [0.45–3.97]	0.6
Male Female				
**Delay between onset of symptoms and admission** ≥ 15 days	8 (8.9) 6 (4.3)	82 (91.1) 134 (65.7)	2.18 [0.73–6.5]	0.08
< 15 days				
**Pallor** YesNo	2 (3.1) 12 (7.3)	63 (96.9) 153 (92.7)	0.4 [0.08–1.86]	0.37
**Splenomegaly** YesNo	13 (6.4) 1 (3.6)	189 (93.6) 27 (96.4)	1.86 [0.23–14.8]	0.55
**Size of splenomegaly** ≥ 2 fingers< 2 fingers	8 (4.8) 6 (9.2)	157 (95.2) 59 (90.8)	0.5 [0.16–1.5]	0.34
**Hepatomegaly**YesNo	5 (4.9) 9 (7)	97 (95.1) 119 (93)	0.68 [0.22–2.1]	0.5
**Nutritional status**HypotrophicEutrophic	1 (2.4) 13 (6.9)	41 (97.6) 175 (93.1)	0.33 [0.02–2.52]	0.47
**Bleeding** YesNo	3 (42.9) 11 (4.9)	4 (57.1) 212 (95.1)	14.5 [2.87–72.7]	0.005
**Co-infections** YesNo	2 (3.9) 12 (6.7)	49 (96.1) 167 (93.3)	0.57 [0.12–2.62]	0.74
**Leucopenia** Yes No	8 (8.6) 6 (4.4)	85 (91.4) 130 (95.6)	2.04 [0.68–6.08]	0.19
**Haemoglobin (g/dL)** < 6≥ 6	7 (14) 7 (3.9)	43 (86) 173 (96.1)	4 [1.33–12]	0.022
**Platelets counts (/mm**^**3**^**)** < 50,000≥ 50,000	7 (14.9) 7 (3.8)	40 (85.1) 176 (96.2)	4.37 [1.45–13.2]	0.011
**Cytolysis** Yes No	6 (27.3) 5 (2.7)	16 (72.7) 181 (81)	13.6 [3.73–49.5]	<10^−3^
**Hemophagocytic syndrome** YesNo	8 (13.6) 6 (3.5)	51 (86.4) 165 (96.5)	4.31 [1.43–13]	0.014

OR: Odds Ratio; CI: Confidential Interval

However, delay between onset of symptoms and admission ≥ 15 days (adjusted OR = 11.52, 95% CI [1.58–83.96], p-value = 0.016), leucopenia (adjusted OR = 9, 95% CI [1.43–56.63], p-value = 0.019), bleeding (adjusted OR = 24.38, 95% CI [1.85–321.75], p-value = 0.015) and cytolysis (adjusted OR = 28.13, 95% CI [4.55–173.6], p-value <10–3), were identified by multivariate logistic regression as independent risk factors for mortality in children with ZVL (Table 3).

**Table 3:** Multivariate analysis of risk factors of mortality in children with ZVL.

Factor	Adjusted OR	95%CI	p
Delay between onset of symptoms and admission ≥ 15 days	11.52	[1.58–83.96]	0.016
Leucopenia	9	[1.43–56.63]	0.019
Bleeding	24.38	[1.85–321.75]	0.015
Cytolysis	28.13	[4.55–173.6]	<10^−3^

OR: Odds Ratio; CI: Confidential Interval

**Fig 1:**
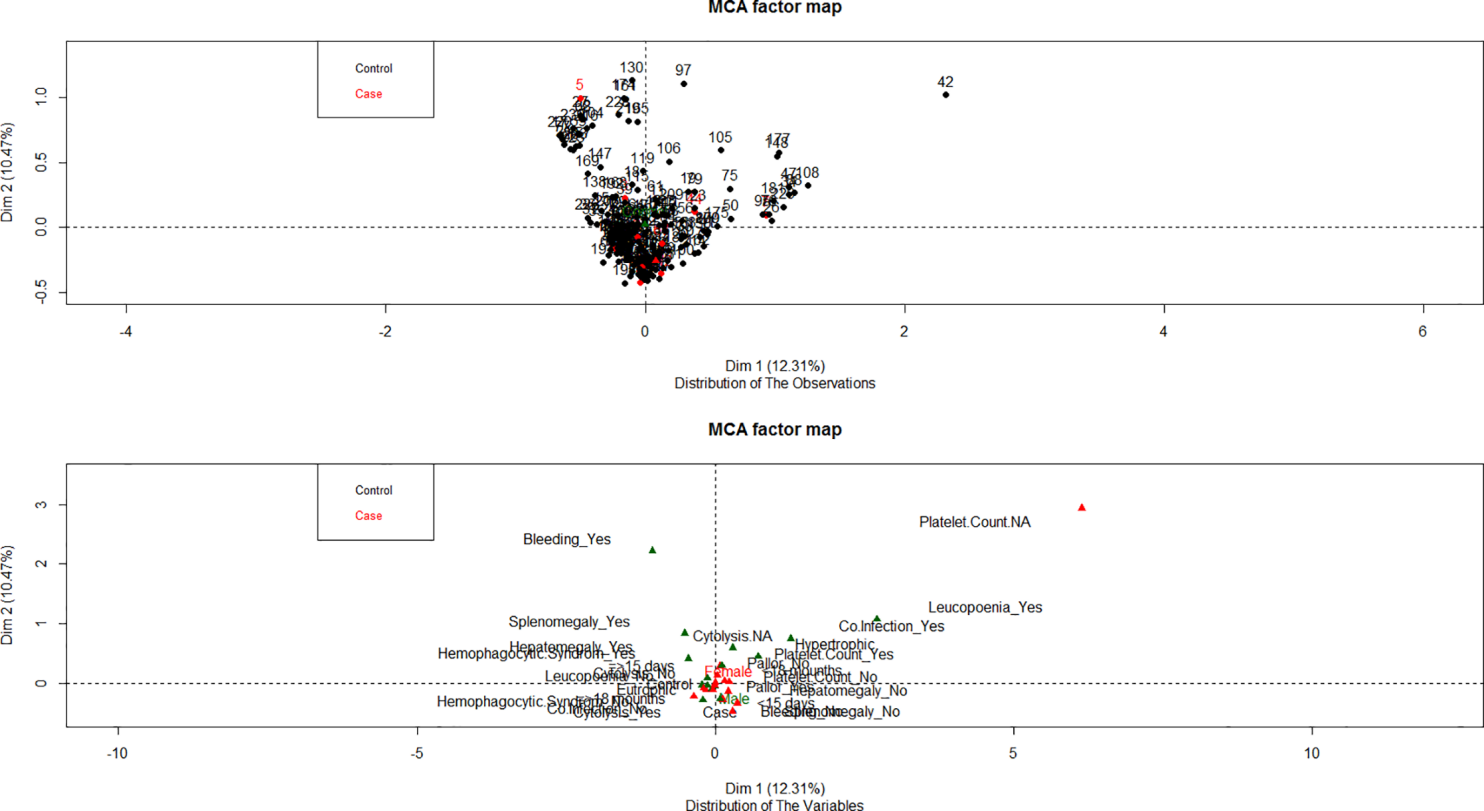
Representation of the categorized clinical data and variables versus morbidity in 2 dimensions resulting from the MCA, in order to describe potential grouping either for individual observations or variables.

On the other hand the MCA did not show a clear correspondence between the categorized clinical variables and the mortality from ZVL (Fig 1). However, for the same data other association between the clinical variables themselves can be seen. For example, an association between splenomegaly, hepatomegaly and platelet count < 50,000. The distribution of the cases (mortality) seems not to be associated to a single clinical variable.

## Discussion

Mortality from ZVL in the pediatric environment remains significant and varies according to countries and health care system access throughout the world from 3.3 to 10% [26–28]. Few studies have examined the factors denoting a poor prognosis of ZVL in children [26, 27, 29, 30]. In our study, delay between onset of symptoms and admission ≥ 15 days, bleeding, leucopenia and cytolysis were considered as independent risk factors of mortality in Tunisian children with ZVL.

Prognostic factors for ZVL in the child population were previously assessed in north Tunisia [29]. In this study, visit delayed more than 56 days, fever lasting more than 21 days, normal or low temperature, haemorrhagic syndrome, haemoglobin rate < 5.5 g/dL, sedimentation rate >25 mm and hypoalbuminemia <30 g/L were identified as poor prognostic factors. In accordance with this study, we found that only the delay between onset of symptoms and admission ≥ 15 days and especially bleeding were identified by logistic regression as significant independent risk factors for mortality in children with ZVL.

Despite the fact that 74% of our study population was under 15 months, we did not find that age carried a worse prognosis, as reported in a previous Tunisian study [29]. Increased risk of poor evolution was reported in children younger than 6 months [31], 12 months [27, 32], 18 months [30], 5 years [33] and even 6 years [34]. The difference between these studies could be explained by the diversity of samples size and particularly the number of patients recruited and the age distribution.

Several studies performed in Latin American and in Africa reported that clinical symptoms at admission such as jaundice, splenomegaly, haemorrhagic syndrome, malnutrition and dyspnoea are considered as factors related to death or severity of ZVL [26, 27, 30, 33, 35, 36]. Scoring system suggests that these clinical manifestations could predict ZVL mortality and contribute to better clinical management [31]. However, our results showed that only bleeding is strongly associated with mortality. The detection of bleeding upon admission or during the course of treatment is crucial in the identification of severity [29, 33]. Haemorrhage was described as the consequence of a ZVL induced inflammatory process with a cascade of events comprising activation of the inflammatory response, development of endothelial lesions, activation of intravascular clotting, hypoperfusion, hypoxaemia and cell death [37].

As reported in some Brazilian studies [26, 30], our results showed that severe anaemia (haemoglobin <6 g/dL) is not associated with increased mortality. However, this finding is not in accordance with studies performed in Tunisia [29], and in Sudan [36, 38].

Despite the fact that platelet counts less than 50,000 cells/ mm^3^ and even less than 85,000 cells/ mm^3^ have been described as poor prognostic factors in Brazilian children with ZVL [26,30], the Leishmaniasis Surveillance Program of the Brazilian Ministry of Health recommended hospitalization for patients with platelet counts <50,000 /mm^3^. However, we found no association between platelet counts <50,000 /mm^3^ (which was observed in 20.4% of patients) in multivariate model and fatality. It was concluded that rather than attempting to define a standard limit of thrombocytopenia, it is more important to assess each case separately in order to decide about the most appropriate hemotherapeutic approach [33].

While neutropenia constituted a predictor for ZVL severity [26, 31, 33], patients with these conditions were probably more susceptible to bacterial infections [33]. Our results showed that a white cell count less than 4,000/mm^3^ is a poor prognostic factor for Tunisian children suffering from ZVL, and therefore, antibiotherapy is highly recommended. While AST and ALT constituted prognosis factors of intermediary evidence in the prediction of poor prognosis [33], we found out that these variables were strongly associated with death of Tunisian children. Increased levels of liver enzymes in patients at admission may signal the presence of hepatitis caused by *Leishmania* infection [39]. Associated bacterial co-infection increases the risk of death from ZVL both in children and adults [26, 33, 35,40]. Only 22.2% of our patients suffered from bacterial co-infection but without influencing their prognosis. It is important to point out the several clinical and biological data are missing. As a mono-center study with a limited sample size, several parameters that can directly influence mortality have not been evaluated including kidney function, liver function and drug toxicity.

In conclusion, our study confirmed the association between death and bleeding and delay of diagnosis in Tunisian children with ZVL. Furthermore, cytolysis and leucopenia must be considered in the management of this pathology.

## Supporting information

S1 Fig Certificate from the Ethical Review Board of the Regional Directory of Health of the governorate of Kairouan. (PDF)

S2 Fig Informed consent. (DOCX)
