# Supplementary material for: Risk factors for mortality of children with zoonotic visceral leishmaniasis in Central Tunisia
Source: PLoS One. 2017 Dec 29;12(12):e0189725. doi: 10.1371/journal.pone.0189725 (PMC5747430; doi:10.1371/journal.pone.0189725)
Supplement: S1 Fig — (PDF) [file pone.0189725.s001.pdf]

Sahloul University Hospital **Ethics Committee**

Sousse, January 02, 2016

Certification

I, undersigned Professor Abdelatif Achour, President of Ethics Committee of Sahloul University Hospital (Sousse, Tunisia), approve the protocol of the study titled "Risk factors for mortality of children with Zoonotic Visceral Leishmaniasis in Central Tunisia".

President of the Committee

Pr. Abdelatif Achour

Hôpital Sahloul de Sousse
Pr Achour Abdellatif
Président Comité d'Ethique
