# Supplementary material for: Risk factors for mortality of children with zoonotic visceral leishmaniasis in Central Tunisia
Source: PLoS One. 2017 Dec 29;12(12):e0189725. doi: 10.1371/journal.pone.0189725 (PMC5747430; doi:10.1371/journal.pone.0189725)
Supplement: S2 Fig — (DOCX) [file pone.0189725.s002.docx]

القيروان في....................

**ترخيص أبوي**

إنني الممضى أسفله (أب) (أم) الطفل.................................... ارخص للفريق الطبي لقسم الأطفال بالقيروان استعمال كل المعلومات الموجودة بالملف الطبي لابني المذكور أعلاه من اجل القيام بدراسات علمية من دون ذكر اسمه .

والسلام.

الإمضاء
